# Diagnostic Performance of ^99m^Tc-Methoxy-Isobuty-Isonitrile (MIBI) for Risk Stratification of Hypofunctioning Thyroid Nodules: A European Multicenter Study

**DOI:** 10.3390/diagnostics12061358

**Published:** 2022-05-31

**Authors:** Simone Agnes Schenke, Alfredo Campennì, Murat Tuncel, Gianluca Bottoni, Sait Sager, Tatjana Bogovic Crncic, Damir Rozic, Rainer Görges, Pinar Pelin Özcan, Daniel Groener, Hubertus Hautzel, Rigobert Klett, Michael Christoph Kreissl, Luca Giovanella

**Affiliations:** 1 Clinic of Nuclear Medicine Bayreuth Hospital, Preuschwitzer Straße 101, 95445 Bayreuth, Germany; 2 Division of Nuclear Medicine, Department of Radiology and Nuclear Medicine, University Hospital Magdeburg, Leipziger Straße 44, 39120 Magdeburg, Germany; michael.kreissl@med.ovgu.de; 3 Nuclear Medicine Unit, Department of Biomedical and Dental Sciences and Morpho-Functional Imaging, University of Messina, Via Consolare Valeria, 98125 Messina, Italy; acampenni@unime.it; 4 Department of Nuclear Medicine, Hacettepe University, Sihhiye, Ankara 06230, Turkey; murat.tuncel@hacettepe.edu.tr; 5 Department of Nuclear Medicine, E.O. “Ospedali Galliera”, 16128 Genoa, Italy; gianluca.bottoni@galliera.it; 6 Department of Nuclear Medicine, Medical Faculty, Istanbul University-Cerrahpaşa, Koca Mustafapasa Caddesi No: 53, Fatih, Istanbul 34096, Turkey; saitsager@yahoo.com; 7 Department of Nuclear Medicine, Faculty of Medicine, University of Rijeka, Brace Branchetta 20/1, 51 000 Rijeka, Croatia; tatjanabc@medri.uniri.hr; 8 Department of Nuclear Medicine, University Hospital Mostar, Bijeli Brijeg bb, 88000 Mostar, Bosnia and Herzegovina; d_rozic@yahoo.com; 9 Department of Nuclear Medicine, University Hospital Essen, Hufelandstraße 55, 45147 Essen, Germany; rainer.goerges@uni-due.de; 10 Department of Nuclear Medicine, University Hospital Mersin, Ciftlikkoy District, MEU Ciftlikkoy Campus, Yenisehir, Mersin 33110, Turkey; ppelinozcan@gmail.com; 11 Department of Nuclear Medicine, University Hospital Frankfurt, Goethe University, Theodor-Stern-Kai 7, 60596 Frankfurt, Germany; daniel.groener@kgu.de; 12 Department of Nuclear Medicine, University Hospital Essen & German Cancer Consortium (DKTK), Hufelandstraße 55, 45147 Essen, Germany; hubertus.hautzel@uk-essen.de; 13 Practice of Nuclear Medicine Hanau/Giessen/Frankfurt/Offenbach, Paul-Zipp-Straße 171, 35398 Giessen, Germany; rigobert.klett@radiol.med.uni-giessen.de; 14 Clinic for Nuclear Medicine and Competence Center for Thyroid Diseases, Imaging Institute of Southern Switzerland, Via Officina 3, 6500 Bellinzona, Switzerland; luca.giovanella@eoc.ch

**Keywords:** ^99m^Tc-MIBI imaging, thyroid scintigraphy, hypofunctioning thyroid nodules, thyroid nodules’ assessment, molecular imaging, risk stratification

## Abstract

^99m^Tc-MIBI (MIBI) imaging is able to exclude malignancy of hypofunctioning thyroid nodules (TNs) with high probability but false positive results are frequent due to low specificity. Therefore, pre-test selection of appropriate TNs is crucial. For image evaluation visual and semiquantitative methods (Washout index, WOInd) are used. Aim of this study was to evaluate the diagnostic performance of MIBI imaging in hypofunctioning TNs with indeterminate fine-needle aspiration cytology results in a multicentric European setting. Patients with hypofunctioning TNs, EU-TIRADS 4 or 5, Bethesda III/IV and MIBI imaging were included. For visual evaluation the intensity of MIBI uptake in the TN was compared to normal thyroid tissue. 358 patients with 365 TNs (*n* = 68 malignant) were included. Planar imaging (SPECT) showed a sensitivity, specificity, positive predictive value, negative predictive value, and accuracy of 96% (94%), 21% (22%), 22% (15%), 96% (96%), and 35% (32%). The WOInd (38.9% of all cases, optimal cutoff: −19%) showed a sens 100% (spec 89%, PPV 82%, NPV 100%, ACC 93%). For hypofunctioning TNs at intermediate or high risk with indeterminate cytology, a MIBI negative result on visual evaluation is an effective tool to rule-out thyroid malignancy. The semi-quantitative method could considerably improve overall diagnostic performance of MIBI imaging.

## 1. Introduction

Since the 1990s, many studies have been published, showing that imaging with ^99m^Tc-labelled Methoxy-isobutyl-isonitrile (MIBI) is a suitable tool to rule-out thyroid malignancy in the diagnostic algorithm due to its high negative predictive value (NPV). In contrast, the specificity and the positive predictive value (PPV) were reported to be low, resulting in a suboptimal overall accuracy (ACC) [1,2,3,4,5]. 

Although the EANM practice guidelines/SNMMI procedure standards for RAIU and thyroid scintigraphy described the protocols and interpretation criteria [6], no standardized MIBI imaging guideline with harmonized imaging interpretation criteria exists to compare clinical results or to improve diagnostic accuracy, respectively. Data have been published that demonstrated superiority of a semi-quantitative approach (washout index, WOind) [1]. However, a recently published European survey showed that various methods of visual image interpretation are still preferred by most centers [7]. These methods are characterized by comparing the MIBI uptake in the thyroid nodule (TN) to the uptake in the adjacent normal thyroid tissue or to the ^99m^Tc-pertechnetate uptake, respectively. In contrast, the semi-quantitative approach takes into account the washout kinetics of MIBI from the early to the late image time-point using regions-of-interest (ROIs) [8,9]. In addition, by selecting the appropriate TNs for MIBI imaging, a higher pretest probability could probably be achieved resulting in an increase of overall diagnostic accuracy. 

The first diagnostic step for assessing TNs is thyroid ultrasound. It estimates the risk of malignancy depending on the presence of suspicious ultrasound criteria by using risk stratification systems such as EU-TIRADS. Low-risk TNs can safely be followed by ultrasound, whereas intermediate- and high-risk TNs require further diagnostic workup [10]. For TNs of ≥10 mm in maximum diameter, ^99m^Tc-pertechnetate scintigraphy can be performed to identify hyperfunctioning TNs which are presumed to be benign with a very high probability [6]. In 2013, Treglia et al. showed in their meta-analysis, that the specificity and overall diagnostic accuracy of MIBI imaging can be improved by excluding hyperfunctioning TNs [3]. Finally, fine-needle aspiration cytology (FNAC) is the most widely used diagnostic test for TNs; it has a high accuracy rate. A benign result has a NPV of >95% and a result suspicious for malignancy has a PPV of 99%. However, the diagnosis of a follicular malignancy is not possible because the capsular and/or vascular invasion has to be verified using the surgical specimen [11]. Furthermore, up to 30% of the FNAC results are indeterminate. This especially holds true for follicular lesions, of which about 70–80% are benign according to operative pathology [11,12,13]. 

The aim of this multicentric study was to evaluate the diagnostic performance of MIBI imaging and compare the visual and the semi-quantitative interpretation methods in a large multicenter series of patients presenting with hypofunctioning TNs classified as intermediate- or high-risk according to EU-TIRADS and indeterminate results in FNAC. 

## 2. Methods

### 2.1. Patients

This is a retrospective, non-interventional, multicenter study. The local ethics committee of the University Hospital Magdeburg approved the study and the need for an informed consent was waived (RAD No. 378, 32/20). All clinical procedures were routine clinical examinations and conducted according to the Declaration of Helsinki. 

Inclusion criteria were:

*a*  age ≥ 18 years,
*b*  hypofunctioning TN of at least 10 mm in maximum diameter,
*c*  EU-TIRADS risk class 4 (intermediate-risk) or 5 (high-risk) as determined by thyroid ultrasound,
*d*  MIBI imaging and:
  (1) FNAC with indeterminate results (Bethesda classification III, IV, ICCRTC TIR 3A/B/4) with available histopathological results, or
  (2) FNAC with benign results (Bethesda classification II, ICCRTC TIR 2) with either histopathological results or follow-up of at least 12 months without progression (not more than 3 mm growth in one diameter).

We excluded low-risk TNs (EU-TIRADS 3) and incomplete sonographic classification, TNs without size measurement, hyperfunctioning TNs, missing histopathology in case of indeterminate FNAC results and all cases without FNAC in the diagnostic workflow.

### 2.2. Thyroid Ultrasound

Thyroid ultrasound was performed by experienced examiners at each participating study center. Detailed information about the procedures and devices have been published [7]. All TN’s data were retrospectively classified according to EU-TIRADS by one experienced examiner (SAS): cysts or entirely spongiform TNs as well as entirely isoechoic or hyperechoic TNs without suspicious features were classified as low-risk TNs (EU-TIRADS 2 or 3, 0% and 2–4% risk of malignancy). A mildly hypoechoic TN without suspicious features such as non-oval/round shape, irregular margins, microcalcifications was categorized as an intermediate-risk TN (EU-TIRADS 4, risk of malignancy 6–17%). The presence of one or more of the suspicious features resulted in the classification of high-risk category (EU-TIRADS 5, risk of malignancy of 26–87%) [10]. TNs that were classified as EU-TIRADS 3 were considered benign and TNs that were EU-TIRADS 4 or 5 were deemed intermediate and highly suspicious for malignancy, respectively. 

### 2.3. Fine Needle Aspiration Cytology

Detailed information about center-specific indications for FNAC have been described previously [7]. FNAC results were classified at each participating study center according to:

(1) The Bethesda system for reporting thyroid cytology (TBSRTC): class I non-diagnostic, class II benign, class III indeterminate (atypia of undetermined significance/follicular lesion of undetermined significance), class IV follicular neoplasm/suspicious for a follicular neoplasm, class V suspicious for malignancy, and class VI malignant [14].
(2) The Italian consensus for the classification and reporting of thyroid cytology (ICCRTC): TIR I non-diagnostic, TIR 1C non-diagnostic cystic, TIR 2 benign, TIR 3A low-risk indeterminate lesion, TIR 3B high-risk indeterminate lesion, TIR 4 suspicious of malignancy, TIR 5 malignant [15].

### 2.4. MIBI Imaging

Center-specific image acquisition parameter and administered MIBI activities have been previously reported [7]. Early MIBI images were acquired 10 min (median) and late images 60 min (median) after injection of 370 MBq (median) of the tracer. For visual analysis, all participating centers were asked to re-evaluate their late and—if available—early MIBI scans according to the following criteria:

#### MIBI Image Analyses

Visual analysis included evaluation of early and late planar images and SPECT imaging data as well (1) and the MIBI kinetics within TNs was also assessed (2). In some centers, additional semi-quantitative results (WOInd) were available (3).

(1) For both, early and late planar and SPECT images, the visual method described the MIBI uptake in the TN compared to the paranodular thyroid tissue as *hypointense* (uptake TN < uptake in paranodular tissue), *isointense* (uptake TN = uptake paranodular), and *hyperintense* (uptake TN > uptake paranodular). A hypointense MIBI uptake was defined as benign, isointense or hyperintense MIBI uptake was considered suspicious for malignancy [2].
(2) The visual assessment of washout kinetics of MIBI was classified as follows:
  *(i)*  *visual pattern A*: reduced uptake in the nodule in the early and late image,
  *(ii)*  *visual pattern B*: uptake in the nodule that decreases from early to late image,
  *(iii)*  *visual pattern C*: uptake in the nodule that remains unchanged or has further increased on the delayed image.

Pattern C was considered suspicious for malignancy, whereas pattern A and B both were considered indicative for a benign TN [9].

(3) The WOInd quantifies the percentage MIBI uptake reduction in a TN between the early and the late image. A region of interest (ROI) was drawn manually around the TN of interest on the early image (TN early) and then mirrored outside the thyroid gland for subtracting the background activity (background on the early image).

The early result (ER) was calculated:

Mean counts TN early—mean counts background early.

Subsequently, both ROIs were copied onto the late image (TN late, background late). 

Late result (LR) was calculated: 

Mean counts TN late—mean counts background late.

For calculating the washout index, the following formula was used according to Campenni et al. [8]: *WOind* (%) = *LR/ER* × 100 − 100.

### 2.5. Statistics

The statistical analysis was performed using WinSTAT, version 2012.1.0.96, 2017, R. Fitch Software, Bad Krotzingen, Germany and BIAS for Windows, version 11.02-01/2016, Epsilon-Verlag 1989–2016, Goethe-Universität Frankfurt, Germany for the ROC analysis. The descriptive statistical parameters are expressed as the mean ± standard deviation (SD) or median and interquartile range (25th/75th-Percentile) depending on whether the given parameter showed a normal distribution or not. The Mann-Whitney U-test and *t*-test were used for statistical analyses as indicated. ROC analysis was used to calculate the optimal cutoff for semi-quantitative parameters. Statistical significance was assumed at *p*-values < 0.05.

## 3. Results

We analyzed the data of 12 study centers re-evaluated from December 2019 to December 2020 (*n* = 1430 TNs). We excluded 395 TNs that were classified as EU-TIRADS 3 or less (low-risk) and 28 TNs that were not classifiable according to EU-TIRADS due to missing description of ultrasound criteria. One TN was excluded because it was smaller than 10 mm. In 72 cases, ^99m^Tc-pertechnetate scintigraphy was not performed before MIBI imaging or the TN was not classified as hypofunctioning. Finally, 84 TNs were excluded due to missing data (i.e., size measurement, sonographic criteria, functional status). 

Overall, 284 females (53.5 ± 13.3 years) and 74 males (53.2 ± 12.6 years; *p* = 0.618) with 365 TNs (maximum size median 25 mm (19/33 mm), *n* = 297 benign, *n* = 68 malignant) fulfilled all inclusion criteria (Table 1). Benign TNs were slightly smaller (median 25 mm (19/32 mm)) as compared to malignant TNs (median 28 mm (19/34 mm)), but the difference was not statistically significant (*p* = 0.318). Of all benign TNs, 127 (42.8%) were classified as EU 4 (intermediate-risk) and 170 (57.2%) were EU 5 (high-risk). Of the 68 malignant TNs, 58 (85.3%) were EU-TIRADS 5 (high-risk) and 10 (14.7%) were classified as EU 4 (intermediate-risk).

Histopathological results were available for 251 of 365 TN including all 68 malignant TNs. Histopathology revealed 86 cases (47.0% of all benign cases) of multinodular goiter (MNG), 71 (38.8% of all benign cases) follicular adenomas (FAs), 60 (88.2% of all malignant cases) papillary thyroid cancer (PTC), 26 (14.2% of all benign cases) oncocytic adenomas (OAs), 6 (8.8% of all malignant cases) follicular thyroid cancer (FTC), one (1.5% of all malignant cases) poorly differentiated thyroid cancer (PDTC), and one (1.5% of all malignant cases) medullary thyroid cancer (MTC). 

Table 2 provides the results of MIBI imaging (visual analysis) findings in the subgroup of TNs with histopathological results. It shows that the majority of PTC and FTC were visually MIBI positive. 

In the kinetic analysis, no significant differences were found in malignant nodules as most visually MIBI positive malignant nodules showed a reduced MIBI washout from the nodule.

MNG were MIBI positive using the visual methods but showed a high washout. Interestingly, OAs and FAs were not distinguishable by the visual method, but by the WOInd. 

**Figure 1:**
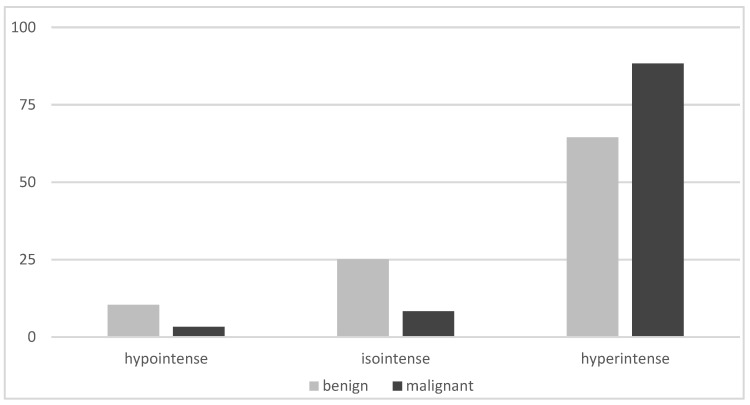
Early planar MIBI imaging results (% of thyroid nodules, MIBI uptake in the thyroid nodule compared to the MIBI uptake in the paranodular tissue).

**Figure 2:**
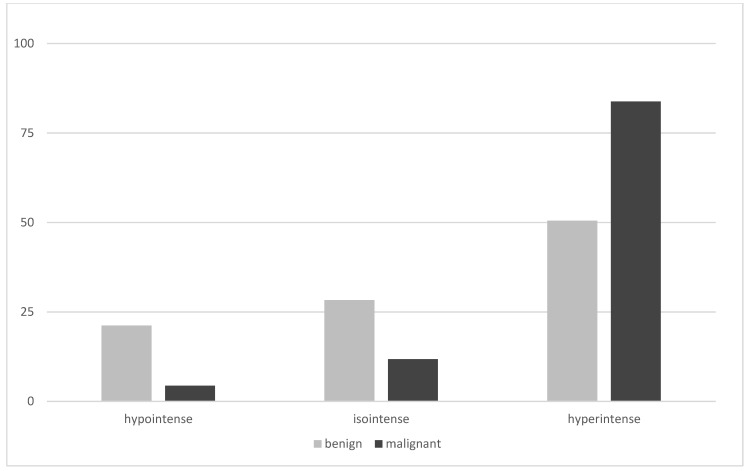
Late planar MIBI imaging results (% of thyroid nodules, MIBI uptake in the thyroid nodule compared to the MIBI uptake in the paranodular tissue).

**Figure 3:**
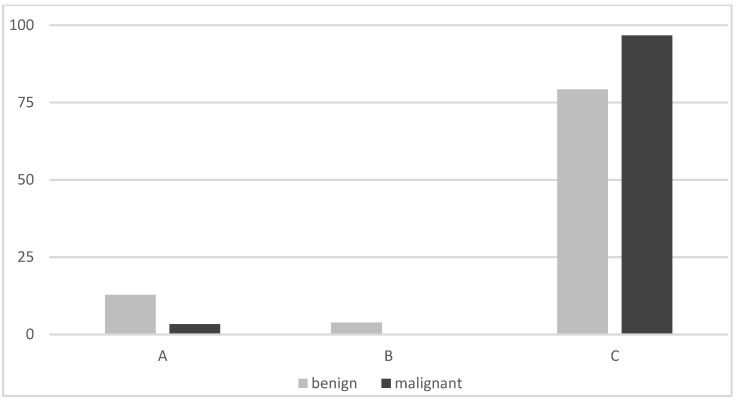
Visual pattern of early and late MIBI uptake (% of thyroid nodules): A: no uptake in the nodule early and late, B: uptake in the nodule that had decreased from early to late image, C: uptake in the nodule that remained unchanged or had further increased on the delayed image.

**Figure 4:**
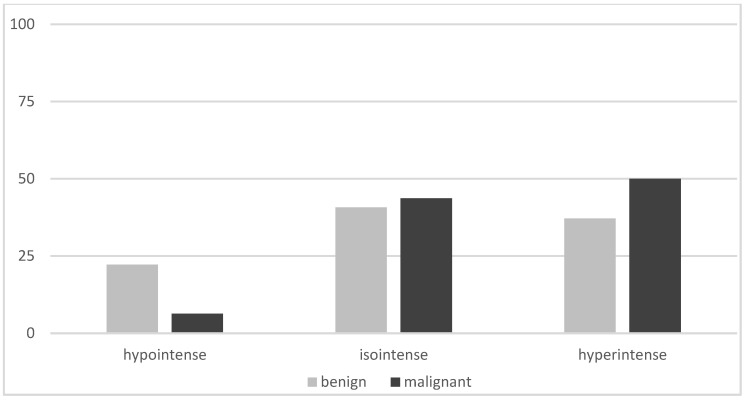
MIBI SPECT results (% of thyroid nodules, MIBI uptake in the thyroid nodule compared to the MIBI uptake in the paranodular tissue).

**Figure 5:**
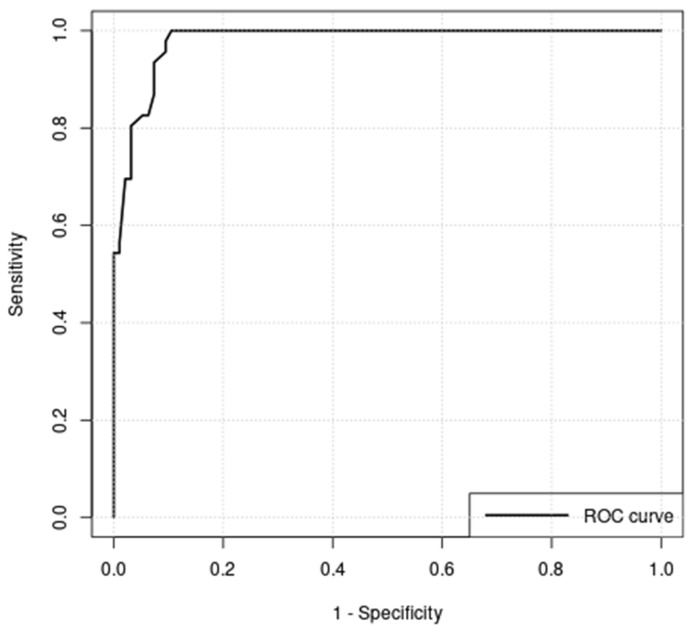
Receiver operating curve analysis of washout index (WOInd); optimal cutoff = −19%; area under curve (AUC) = 0.980.

**Figure 6:**
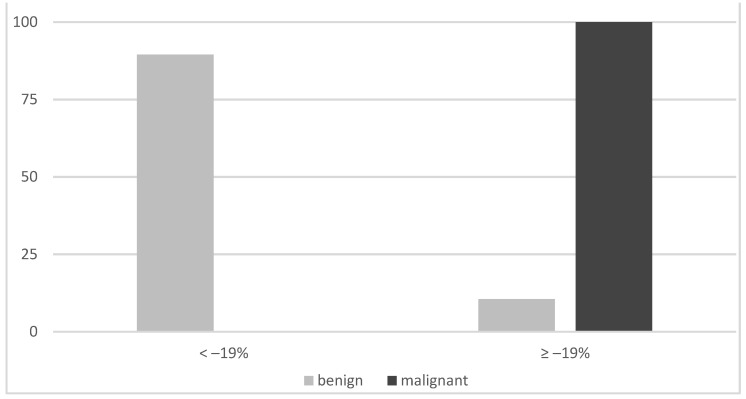
Semiquantitative results (cutoff WOInd −19%).

On planar imaging (i.e., early, late and early to late comparison), about 90% of malignant TNs and 70% of benign TNs were classified as hyperintense compared to the paranodular thyroid tissue (Figure 1, Figure 2 and Figure 3). SPECT images were performed in *n* = 124 (34.0%) of all TNs (Figure 4). A hyperintense SPECT pattern was observed in 8 out of 16 (50.0%) of the malignant and 40 out of 108 (37.0%) benign TNs, respectively. A hypointense SPECT pattern was observed in 24/108 (22.2%) of the benign TNs. The WOind was calculated in a subgroup of *n* = 142 cases (38.9%). The ROC curve analysis was performed to select the WOind optimal cutoff (Figure 5). Adopting the ROC-derived threshold of −19% semiquantitative image analysis performed significantly better than planar and SPECT visual evaluation methods (Figure 6, Table 3). 

## 4. Discussion

**Figure 7:**
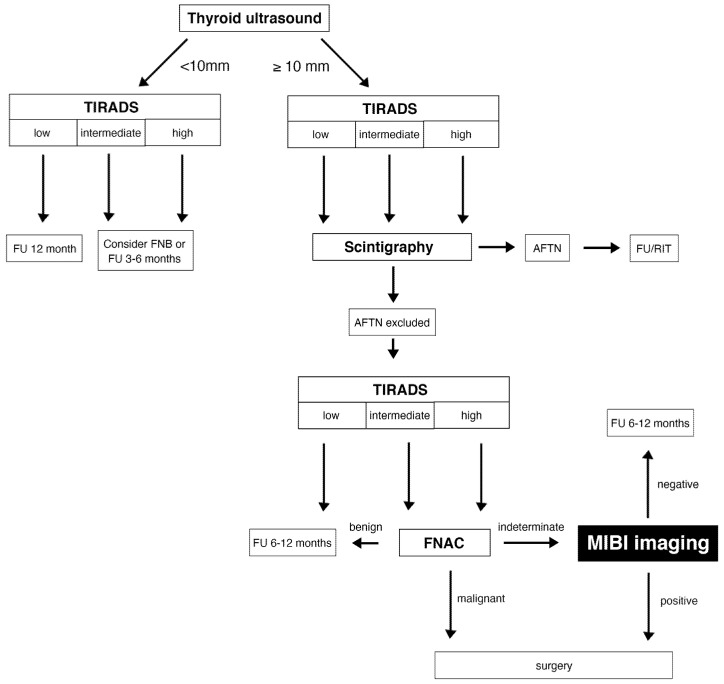
Diagnostic algorithm for the assessment of thyroid nodules. Legend: TIRADS–Thyroid Imaging Reporting and Data System; FU–Follow up; AFTN–Autonomously functioning thyroid nodule; RIT–Radioiodine therapy; FNB -fine-needle biopsy; FNAC–fine-needle aspiration cytology.

Our present study is the first to examine the diagnostic performance of MIBI imaging in a large multicenter series of patients selected using predefined inclusion and exclusion criteria (see Figure 7). 

The high number of benign lesions should be accounted for as it increases the pre-test probability of excluding malignant lesions (high NPV). However, the prevalence of malignancy is 18.6% in our collective, which is equal to or higher than in previous studies [1,4,5] and in line with malignancy rates reported in patients with cytologically indeterminate nodules [14,15].

The main result of our study is, that all examined interpretation criteria of MIBI imaging approaches showed a sensitivity and NPV > 90%, which is well in line with previously published studies [1,2,16,17]. Unfortunately, specificity and PPV were suboptimal using visual approaches making interpreted MIBI imaging unsuitable as rule-in biomarker.

Additionally, the SPECT approach was not superior to the planar late image interpretation in our study. Schenke et al. previously reported an improvement in diagnostic ACC with the additional use of SPECT leading to changes of MIBI classification in approximately 30% of the patients [16]. However, SPECT was acquired only in one third of the patients in this study and the change in further assessment of the TNs was not investigated, which may explain this observed difference. On the other hand, the PPV and the specificity of the SPECT approach was comparable in our study and those of Schenke and co-workers (15.2% and 17.9%, specificity 22.2% and 22.5%), respectively. This observation is likely related to a similar percentage of FAs and OAs in both studies [16]. 

Finally, the WOind was the only method that reached 100% sensitivity and NPV with a significantly higher specificity and PPV compared to visual methods, including SPECT, comparable to two prospective monocentric studies by Campenni et al. and Giovanella and co-workers. With comparable preselection of TNs (including only hypofunctioning TNs with intermediate or high risk of malignancy according to EU-TIRADS that showed indeterminate results on FNAC) they examined visual image evaluation and semi-quantitative methods. Both groups reached higher values for specificity (Campenni: 100%; Giovanella: 96%) and PPV (Campenni: 100%; Giovanella: 88%) compared to previous studies that used the visual method alone. However, the cutoff for the WOInd was lower (−9%) in the study by Giovanella et al. than in the one by Campenni et al. (−19%), which could indicate, that a center-specific WOInd may be helpful [1,4,8,9].

In our study, in the subgroup of TNs with histopathological results, the OAs were MIBI positive in all evaluation methods, but the FAs showed a high MIBI washout in the semi-quantitative approach. This observation should be taken into account when selecting TNs with oncocytic changes on cytology for MIBI imaging. OAs are known to be rich of mitochondria, which are the cellular target structures of MIBI [18,19]. On the other hand, papillary thyroid cancer may be identified by both, the visual and the semi-quantitative approach. In a previous study, Campenni and colleagues demonstrated, that all diagnostic parameters could be improved to 100% when patients with OAs were excluded from the analysis of the WOInd [20].

Besides the prevalence of cancer, the composition of the study population is a main factor influencing the results of MIBI imaging. Our study cohort contained a high proportion of FAs and OAs, respectively, which is likely attributable to the inclusion criterion of indeterminate cytology. In another study that analyzed MIBI imaging and TIRADS for hypofunctioning TNs, the percentage of both types of adenomas was lower, resulting in a higher specificity (34.8%) and ACC (43%) of the visual interpretation method than in the current study [21]. An earlier study by Saggiorato also confirmed the relationship between MIBI diagnostic performance and the percentage of OAs. The specificity and the PPV for the visual interpretation approach increased dramatically in the subgroup of non-oncocytic lesions to 81% and 73.3% compared to 9.1% and 28.6% in the group with OAs. Furthermore, the additional use of a semi-quantitative approach improved both parameters as well [22]. Another interesting study concerning the histological degree of differentiation and MIBI uptake was published by Kresnik and colleagues in 1997. This was one of the first studies that examined MIBI imaging in TNs (the prevalence of malignancy was comparable to our study). The authors visually evaluated early (30 min after MIBI injection) and late images (120 min after MIBI injection). The clear retention of MIBI in the TNs detected in the delayed images was defined as MIBI positive, whereas a continuous visual washout was described as MIBI negative. They found that all nine microfollicular adenomas, five of ten FAs, four of eight OAs, and five of ten differentiated thyroid carcinomas were MIBI positive and showed a high degree of differentiation (i.e., G1 and G2 in case of thyroid carcinomas). The remaining thyroid carcinomas showed a lower degree of differentiation. Thus, the authors concluded, that MIBI imaging can reflect thyroid nodules’ degree of differentiation but not their potential of malignancy. However, compared to our study cohort, the TNs were not preselected according to ultrasonography features (TIRADS not yet established at that time) and cytology [23].

**Figure 8:**
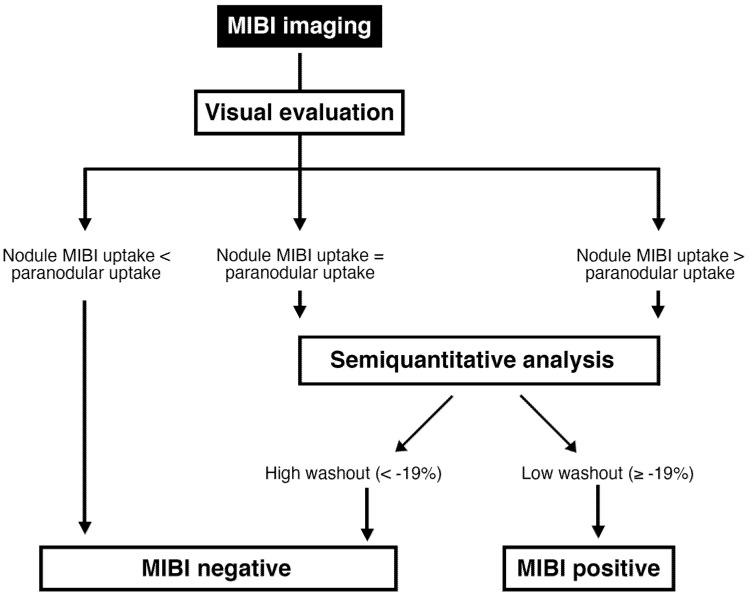
Flowchart for the MIBI image interpretation.

Our study confirmed the high NPV of a MIBI negative result in both, visual and semi-quantitative interpretation method in a European multicentric setting. Therefore, in case of a visually hypointense MIBI uptake or a visual pattern A, TNs can be safely followed up by thyroid ultrasound. On the other hand, if a TN shows an increasing or constant MIBI uptake, an additional semi-quantitative analysis (WOInd) may be recommended (Figure 8).

Due to the retrospective design, our study has some limitations. 

Firstly, the WOInd was only calculated in a limited number of patients and centers, due to the fact that it was no required inclusion criteria of this study. Only 4 of the 12 study centers (Messina *n* = 130, Mostar *n* = 2, Istanbul *n* = 3, and Duisburg *n* = 7) took the semi-quantitative method into account in their diagnostic workflow. Therefore, a prospective multicenter study with standardized acquisition of the WOInd is necessary. On the other hand, our results are well in line with those obtained in previously reported series and consistently confirm superior accuracy of semi-quantitative analysis over visual approaches. Secondly, the varying acquisition protocols used at the different study centers may influence the results of the MIBI imaging. However, most centers applied comparable protocols, as previously reported; therefore, a major impact is not very likely [7]. Thirdly, the EU-TIRADS categories were retrospectively classified according to the documented ultrasound features. This approach could lead to misclassification and therefore may bias the MIBI imaging results. However, all ultrasound image data were collected and evaluated by one expert physician (SAS). This way, interobserver variability and a significant selection bias were avoided. Fourthly, interobserver variability of MIBI imaging was not tested. However, Schenke et al. and Baumgarten et al. showed that the interobserver agreement was good for the planar imaging approach and was improved by using the SPECT technique [24,25]. Finally, our study did not address the cost-effectiveness of MIBI imaging in the clinical setting. Interestingly, higher cost-effectiveness of MIBI imaging compared to repeated FNAC and gene expression classifier was previously demonstrated by Wale et al. and Heinzel et al. [4,12].

## 5. Conclusions

Molecular imaging with ^99m^Tc-MIBI is helpful for the assessment of hypofunctioning thyroid nodules that were classified as intermediate- or high-risk by ultrasound in case of indeterminate thyroid cytology. A MIBI negative result on visual evaluation is an effective tool to rule-out thyroid malignancy. Semi-quantitative image analysis may considerably improve overall diagnostic performance of MIBI imaging but prospective multicentric studies are needed for the confirmation of our results. 

## Figures and Tables

**Table 1:** Patients’ characteristics and acquisition parameters.

	All *n* = 365	Benign TNs *n* = 297	Malignant TNs *n* = 68	*p*-Value
Sex				
Female *n*	284	235	49	
Male *n*	74	55	19	
Age (years ± SD)	53.4 (13.1)	53.7 (12.8)	52.2 (14.9)	0.81
Maximum size (mm)	25	25	28	0.32
Median (25th/75th-p)	(19/33)	(19/32)	(19/34)	
Activity (MBq) *n*	370	370	370	0.13
Median (25th/75th-p)	(323/384)	(314/399)	(370/370)	
Early images (min after injection) *n*	271	211	60	0.001
Median (25th/75th-p)	10 (10/20)	10 (10/20)	10 (10/10)	
Late images (min after injection) *n*	365	297	68	0.12
Median (25th/75th-p)	60 (60/60)	60 (60/60)	60 (60/60)	

TNs—thyroid nodules, *n*—number, SD—standard deviation, mm—millimeter, p—percentile, min—minutes.

**Table 2:** Subgroup of thyroid nodules with available histological results compared to the MIBI imaging methods.

	FA (*n* = 71)	FTC (*n* = 6)	MNG (*n* = 86)	MTC (*n* = 1)	OA (*n* = 26)	PDTC (*n* = 1)	PTC (*n* = 60)
MIBI planar late							
Hypointense (%)	9.9	16.7	23.3	100	7.7	0	1.7
Isointense (%)	8.5	0	19.8	0	0	0	13.3
Hyperintense (%)	81.7	83.3	57	0	92.3	100	85
Visual pattern							
A (%)	3.5	0	17.1	100	5.3	n.a.	1.8
B (%)	3.5	0	4.3	0	0	n.a.	0
C (%)	93	100	78.6	0	94.7	n.a.	98.2
WOInd							
Low washout, ≥−19 (%)	2.4	n.a.	0	n.a.	81.8	n.a.	100
High washout, <−19 (%)	97.6	n.a.	100	n.a.	18.2	n.a.	0

FA—follicular adenoma, FTC—follicular thyroid cancer, MNG—multinodular goiter, MTC–medullary thyroid cancer, OA—oncocytic adenoma, PDTC—poorly differentiated thyroid cancer, PTC—papillary thyroid cancer, WOInd—washout index, n.a.—not applicable.

**Table 3:** Diagnostic performance of visual MIBI imaging evaluation and MIBI washout index.

	Sensitivity (%)	Specificity (%)	PPV(%)	NPV (%)	ACC (%)
MIBI early	96.7	10.4	23.5	91.7	29.5
MIBI late	95.6	21.2	21.7	95.5	35.1
MIBI-SPECT	93.8	22.2	15.2	96	31.5
Visual pattern	96.7	16.6	24.8	94.6	34.3
Washout index cutoff −19%	100	89.4	82.1	100	92.9

SPECT—single photon emission computed tomography, PPV—positive predictive value, NPV—negative predictive value, ACC—accuracy.
